# Mitochondrial Introgression With Potential Functional Effects in North American Yak

**DOI:** 10.1002/ece3.72362

**Published:** 2025-10-18

**Authors:** Leah K. Treffer, Renae L. Schroeder, Edward S. Ricemeyer, Ted Kalbfleisch, Anna M. Fuller, Jessica L. Petersen

**Affiliations:** ^1^ Department of Animal Science University of Nebraska‐Lincoln Lincoln Nebraska USA; ^2^ Department of Biology Nebraska Wesleyan University Lincoln Nebraska USA; ^3^ School of Integrated Plant Science Cornell University Ithaca New York USA; ^4^ Bond Life Sciences Center University of Missouri Columbia Missouri USA; ^5^ Palaeogenomics Group, Institute of Palaeoanatomy, Domestication Research and the History of Veterinary Medicine Ludwig‐Maximilians‐Universität Munich Germany; ^6^ Department of Veterinary Science University of Kentucky Lexington Kentucky USA

**Keywords:** *Bos grunniens*, divergence, introgression, oxidative phosphorylation

## Abstract

The domestic yak ( *Bos grunniens* ) has experienced introgression with domestic cattle in its native Qinghai‐Tibetan Plateau and after introduction to North America (NA), although the extent to which the latter has occurred is not well documented. We used complete mitochondrial (mtDNA) sequences of 12 NA yak and aligned them to the *B. taurus* reference genome for annotation. Identified variation among the NA haplotypes included a total of 982 variants, of which 99 were nonsynonymous single nucleotide polymorphisms. Among the NA yak, we identified nine unique mitotypes, which a haplotype network separated into two distinct clusters. A maximum likelihood tree including 86 Bovidae taxa revealed six NA yak haplotypes formed a clade with *B. indicus* ; the other three haplotypes grouped with *B. grunniens* and fell as a sister clade to bison, gaur, and gayal. These data demonstrate two mitochondrial origins of NA yak, likely dating prior to their importation to NA. We isolated satellite cells from seven yak that represented both major mitotypes ( *B. indicus* [*N* = 4] or yak [*N* = 3]) to investigate possible differences in ATP production. Oxidative consumption rates and extracellular acidification rates were quantified as measures of mitochondrial respiration and glycolysis using the Seahorse ATP Rate Assay. Cells with the *B. grunniens* mitotype had less total energy metabolism (*p* = 0.016). This difference may reflect adaptations to ancestral environments and selective pressures associated with husbandry practices and breeding.

## Graphical abstract

Little is known about the origin of yak that were imported to North America. Using complete mtDNA sequence, we identified two distinct lineages in North American yak; one of these appears to be the consequence of past introgression from indicine cattle. Variation in the mtDNA included many putatively functional variants. In a pilot study of mitochondrial function, we observed a difference in ATP production of satellite cells associated with the mtDNA haplotype.

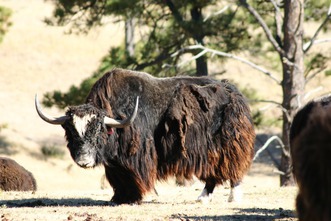

## 1 Introduction

Domestic yak ( *Bos grunniens* ), derived from wild yak ( *Bos mutus* ), are found throughout the Himalayan region of the Indian subcontinent and the Qinghai‐Tibetan Plateau (QTP) that stretches north to Mongolia and Russia. In Asia, the domestic yak is an important economic and cultural bovid. Many ethnic communities use yak for food (milk and meat), shelter (hides), fuel (dry dung), clothing and material (wool), transportation, and in religious ceremonies (Basang et al. 2018; Joshi et al. 2020). Not only have yak remained a central livestock species to pastoralists, but their grazing behaviors are important to the local ecosystem (Jing et al. 2022). The yak is the primary ungulate in the QTP due to its adaptation to the extreme high‐altitude habitat, with limited vegetation, an average elevation of 4000 m above sea level, and an average temperature of −8°C with lows that reach −40°C (Wang et al. 2010; Bailey et al. 2002). Survival of yak in this habitat is thought to be aided by genomic, anatomical, physiological, and behavioral adaptations, as well as by unique rumen microbiota (Qiu et al. 2012; Shi et al. 2018; Ayalew et al. 2021; Jing et al. 2022).

Domestic and wild yak are estimated to have diverged ~7300 years before present, coinciding with glaciation events in the QTP. There are distinct genomic differences between domestic and wild yak indicating that domestication selected for variation in neurologic and behavior‐related genes (Liu et al. 2023). However, there continues to be gene flow between the two species (Qiu et al. 2015). Mitochondrial DNA (mtDNA) analysis is often used to study diversity and population structure. Early studies sequenced only a portion of the yak mitochondrial genome, most commonly the hypervariable D‐loop. As a result, two major lineages of domestic yak were identified (Bai et al. 2014; Shi et al. 2018; Guo et al. 2006; Basang et al. 2018). A comparison of complete mitochondrial genomes (Wang et al. 2010) provided a more comprehensive analysis of yak lineages in the QTP, describing 34 haplotypes in two highly divergent lineages that included both domestic and wild yak with a third lineage comprised only of wild yak. This result was supported by additional mtDNA sequences in Wang et al. (2021, 2024) with these additional analyses finding domestic yak grouping into the third branch with wild yak. Supporting mtDNA data, analysis using whole genome sequences of Asian wild and domestic yak found three genetic groups of domestic yak, developed from one region of origin in the QTP and having had frequent genetic exchange with wild yak that coincided with human movement during the time period of domestication (Chai et al. 2020).

**FIGURE 1:**
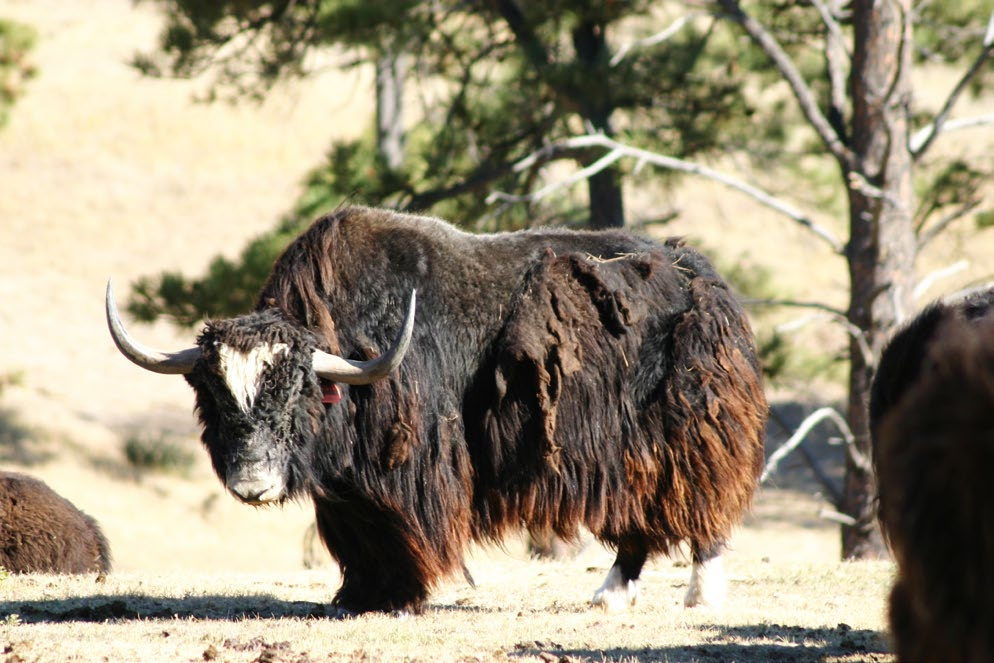
A North American yak bull. Photo by Sheron Grossmann.

Importation of domestic yak to North America (NA) dates to the early 1900s (Brower 2008; Figure 1). Few introductions occurred, so it is assumed to have caused a genetic bottleneck. Pedigrees of the animals imported at that time were not well documented. Consequently, much remains unknown about the lineages of present‐day NA yak, such as from which ancestral population(s) they originated. The earliest imported yak were, in part, intended for hybridization with NA cattle to produce hybrids more tolerant of harsh environments (Wiener et al. 2006). As F1 male hybrids of cattle and yak are infertile, gene flow in a backcross is restricted to contributions of F1 females, which can cross with either cattle or yak bulls (Niayale et al. 2021).

A recent haplotype‐resolved genome assembly of a NA yak (Rice et al. 2020) represented a significant improvement in the continuity of the prior yak reference genome and revealed cattle introgression in the NA yak studied. Based upon those data, a novel tri‐allelic SNP panel (87 markers) was designed to identify recent cattle introgression in NA yak (Kalbfleisch et al. 2020). In that study, it was found that 5%–7.8% of the 170 NA yak studied had cattle ancestry (Kalbfleisch et al. 2020). Although the assay was built to confidently identify recent introgression (e.g., within four generations), the overall frequency of cattle alleles identified was low (0.004), and the minimal signature of introgression was attributed to introgression that likely took place prior to importation into NA. Ancestral introgression with cattle is reported in Asian yak, although those yak are genetically and physiologically different from Asian cattle (Jacques et al. 2021).

Genomic information can clarify the lineages of NA yak. Understanding that most NA yak have few cattle alleles in the autosomal genome (Kalbfleisch et al. 2020), we predicted clear differences in mtDNA between cattle and NA yak. Data from the NA yak genome assembly and prior work (Treffer et al. 2020) established a need to examine the mitochondrial genome of NA yak, which previously has not been well characterized. The present study therefore aimed to analyze NA yak diversity using the complete mtDNA sequence. The characterization of NA yak lineages can contribute to the management of the genetic diversity of these herds. Additionally, physiological adaptations play a part in the ability of yak to survive in harsh conditions. Due to the role of mitochondria in cellular oxygen consumption and cellular energy production, this project also aimed to identify functional differences in the mitochondrial genome of the NA yak, studied by measuring cellular respiration of NA yak skeletal muscle (satellite cell) isolates. We hypothesized that mitochondrial variants altering protein composition and differentiating mtDNA lineages would result in functional differences in cellular metabolism.

## 2 Materials and Methods

### 2.1 Samples

Whole‐genome sequence (WGS) was generated from 13 NA yak, described in Rice et al. (2020). These animals include the individual from which the yak reference was derived (ARS_UNL_BGru_maternal_1.0 Mitochondrion MT) and were selected based on recent pedigree records with the intention of representing diversity in the NA population. Sequences were aligned to *Bos taurus* ARS‐UCD1.3 with BWA‐MEM ver 0.7 (Li 2013). One sequence was suspected to be either contaminated or heteroplasmic and was removed from this study. Mitochondrial sequences were extracted from the BAM file and converted to fasta using samtools (Li et al. 2009).

### 2.2 NA Yak Variants and Mitotypes

Variants were identified and manually annotated in MEGA X: Molecular Evolutionary Genetics Analysis across computing platforms (Kumar et al. 2018). Sequences in fasta format were aligned using MUSCLE Multiple Sequence Alignment feature (Edgar 2004) based upon Ensembl annotation v114 and exported as a nexus file. Variants identified between NA yak and the reference, and among NA yak haplotypes were recorded. Variant information was uploaded into the Variant Effect Predictor (VEP) web interface (McLaren et al. 2016) and compared to the cattle reference (ARS‐UCD1.3) Ensembl transcripts database to determine the predicted impact of each variant on protein function.

### 2.3 NA Yak Mitotypes Excluding the D‐Loop

Due to its variability, the D‐loop (CM008198.1:m.1_363 and CM008198.1:m.15792_16109) was removed from the 12 NA yak sequences. Haplotypes were identified from the truncated fasta alignment using the *pegas* v1.1 (Paradis 2010) package in Rstudio 4.1.0.

### 2.4 Phylogenetic Trees Using Complete MT Genome

For analysis of how the NA yak relates to Asian domestic yak, wild yak, cattle, and other Bovidae, dendrograms were created utilizing all unique NA yak mitochondrial haplotypes (*N* = 9) and 74 other Bovidae complete mitochondrial sequences downloaded from NCBI GenBank (Benson et al. 2013) (Table S1) as fasta files. The mitochondrial genome from the *Ovis aries* reference genome (ARS‐UI_Ramb_v3.0) was used as the outgroup (NC_001941.1). Among the Asian domestic yak downloaded from GenBank were accessions that represent each of the lineages and haplotypes described by Wang et al. (2021) (GQ464257.1, GQ464251.1, GQ464260.1, GQ464279.1, GQ464266.1), which were aligned to the above taxa in MEGA X as described above. The alignment was then converted to a phylip file in Geneious Prime 2020.0.5 (https://www.geneious.com).

The alignment in phylip format was modeled with PartitionFinder2.1.1 (Lanfear et al. 2017) with RAxML (Stamatakis 2006). Input for Partition Finder was the aligned taxa file in addition to a configuration file [branchlengths = linked, models = all, model selection = aicc, search = rcluster (Lanfear et al. 2014)]. The data block of codon positions was identified using the cattle Ensembl annotation v108.12. The Partition Finder analysis determined the best scheme, which was automatically formatted into nexus character sets for IQtree.

The phylip file of aligned taxa along with the nexus partition file was submitted to IQtree7 (Trifinopoulos et al. 2016) for tree inference. Maximum likelihood trees were produced with branch support evaluated with 1000 bootstrap iterations. Further visualization and annotation of the tree were done by downloading the maximum likelihood tree in newick format from IQtree and reading into Rstudio v 4.3.0 using the *ggtree* package (Xu et al. 2022).

Separate from the above analyses, more recent complete mtDNA sequence representing 21 breeds of Asian yak was obtained from Wang et al. (2024). One representative sequence (fasta) of each of the 278 haplotypes identified was aligned to the NA yak after removal of the D‐loop.

### 2.5 Domestic Yak Haplotype Network (D‐Loop)

To allow for a greater comparison with existing data, D‐loop sequences of the NA yak were categorized in their relationship to the established lineages of the Asian yak. Publicly available sequences of 29 domestic yak D‐loop regions from previous studies were downloaded from GenBank (Table S1) as fasta files, the NA sequences were manually truncated to the same 835 bp fragment, and aligned with MEGAX as described above. The D‐loop sequences were imported into PopART (Leigh et al. 2015) as a nexus file, and a median joining network was created with a 0 epsilon parameter.

### 2.6 Sanger Sequencing

We used Sanger sequencing of 17 additional NA yak to determine if there was additional variation in the population using five primer pairs designed to cover 3521 bp. Primers were designed using the ARS‐UCD1.3 cattle reference and methods described in Supporting Information. Resulting chromatograms were visualized in Sequencer v 5.4.6 (Gene Codes, Ann Arbor, MI).

### 2.7 Cell Isolation and Culture

Skeletal muscle was collected from seven domestic yak harvested at privately owned abattoirs and shipped overnight on ice in Belzer UW Cold Storage Solution (Global Transplant Solutions, Spartanburg, SC). DNA from each individual, isolated following the protocol outlined in Reith et al. (2024) was sent to Neogen (Lincoln, NE) for their commercial genotyping test to estimate cattle introgression (Kalbfleisch et al. 2020), and for the classification of mitochondrial genotype. The classification of mitotype (cattle vs. yak) was additionally verified by Sanger sequencing of the *ATP6* locus, the marker used in the commercial genotyping panel.

Primary satellite cells were isolated using the protocol described by Sieck et al. (2022). In brief, primary satellite cells were isolated via protease digestion and serial centrifugation, then suspended in complete growth medium (78.5% DMEM, 20% fetal bovine serum, 1% AbAm [Thermo‐Fisher Scientific, Waltham, MA], and 0.5% gentamycin [Thermo‐Fisher Scientific, Waltham, MA]). Cells were seeded onto poly‐l‐lysine and fibronectin coated cell culture plates and were incubated at 37°C and 5% CO_2_.

The thawing procedure followed Posont et al. (2022); myoblasts were quickly thawed at 37°C and pre‐plated in DMEM containing 10% FBS for 2 h (37°C; 95% O_2_, 5% CO_2_). They were then expanded for 48 h before being plated in six‐well tissue culture plates for functional experiments.

To determine the purity of myoblast isolates, a subsample of 50,000 cells from each isolate was stained for the myoblast marker pax7 (mouse anti‐pax7, 1:50; Abcam, Cambridge, MA, USA) as previously described (Yates et al. 2014).

As sample collection took place at two different abattoirs and the muscle sampled was not recorded, a Western blot was used to identify muscle fiber type proportions to ensure samples were not divergent in fiber composition (Supporting Information).

### 2.8 ATP Rate Assay (Seahorse ATP Rate Assay Kit)

Cultured cells were thawed, suspended in complete growth media, seeded onto a poly‐l‐lysine and fibronectin coated Seahorse Cell Culture Microplate (Agilent, Santa Clara, CA) at a density of 100,000 cells/well, and incubated at 37°C and 5% CO_2_ overnight. The injection plate was hydrated with 1 mL seahorse calibrant overnight. The cultured cells were washed with Seahorse XF Assay Media supplemented with 100 mM sodium pyruvate, 200 mM glutamine, and 2 M glucose and incubated at 37°C for 1 h. The injection stock reagents were diluted with supplemented Seahorse XF Assay Media (Oligomycin 100 μM and Rotenone 50 μM) then added to the injection ports. Real‐time measurements of mitochondrial oxygen consumption rates (OCR) and glycolytic extracellular acidification rates (ECAR) were collected at 15 time points with the ATP Rate Assay on a Seahorse XFe24 Extracellular Flux Analyzer to evaluate the metabolic pathways being used by the cells (Agilent Seahorse XF Real‐Time ATP Rate Assay Kit User Guide).

After the assay was run, protein was extracted with 25 μL M‐PER Mammalian Protein Extraction Reagent (Thermo Fisher Scientific) per well. Twenty microliters of the M‐PER cell mixture was then dyed with 1 mL of Pierce dye from a Pierce Coomassie Plus Assay Kit (Thermo Fisher Scientific). Absorbance was measured using a spectrophotometer, protein concentrations were calculated, and these data were utilized for normalization of OCR and ECAR in the Seahorse Wave desktop software.

Outlier well and individual time‐point measurements were excluded based on the mean average deviation as previously described (Yépez et al. 2018). Technical replicates for each cell isolate were averaged. Cell isolates were run on two assay plates, with one cell isolate serving as a control between both plates. Intracellular flux analyses can result in inter‐plate variation (Yépez et al. 2018). To mitigate this, the ATP production rates for the second plate were proportionally scaled so that the total ATP production rate for the cell isolate repeated on both plates was equal. Differences in ATP production due to genotype (cattle vs. yak) were evaluated via ANOVA using the Mixed Procedure with repeated measures (technical replicates of each animal) in SAS (SAS Institute, Cary NC). The appropriate covariance structure was selected based on best‐fit statistics, and plate was included as a covariate.

## 3 Results

### 3.1 NA Yak Mitotypes

The complete mtDNA sequence of the 12 NA yak contained 16,709 bp. Relative to the cattle reference genome, 1116 variants were identified. When only considering the NA yak mitotypes (excluding variation fixed in all yak relative to the reference), 982 variants were present (Table S2). These variable sites were captured within nine unique mitotypes; eight were represented by individual animals and one mitotype was shared by four yak. Excluding the D‐loop, three unique mitotypes were present among the 12 NA yak. One was present in seven NA yak, the second in three NA yak, and the third was shared by two NA yak.

### 3.2 Phylogenetic Tree‐Complete MT Genome

**FIGURE 2:**
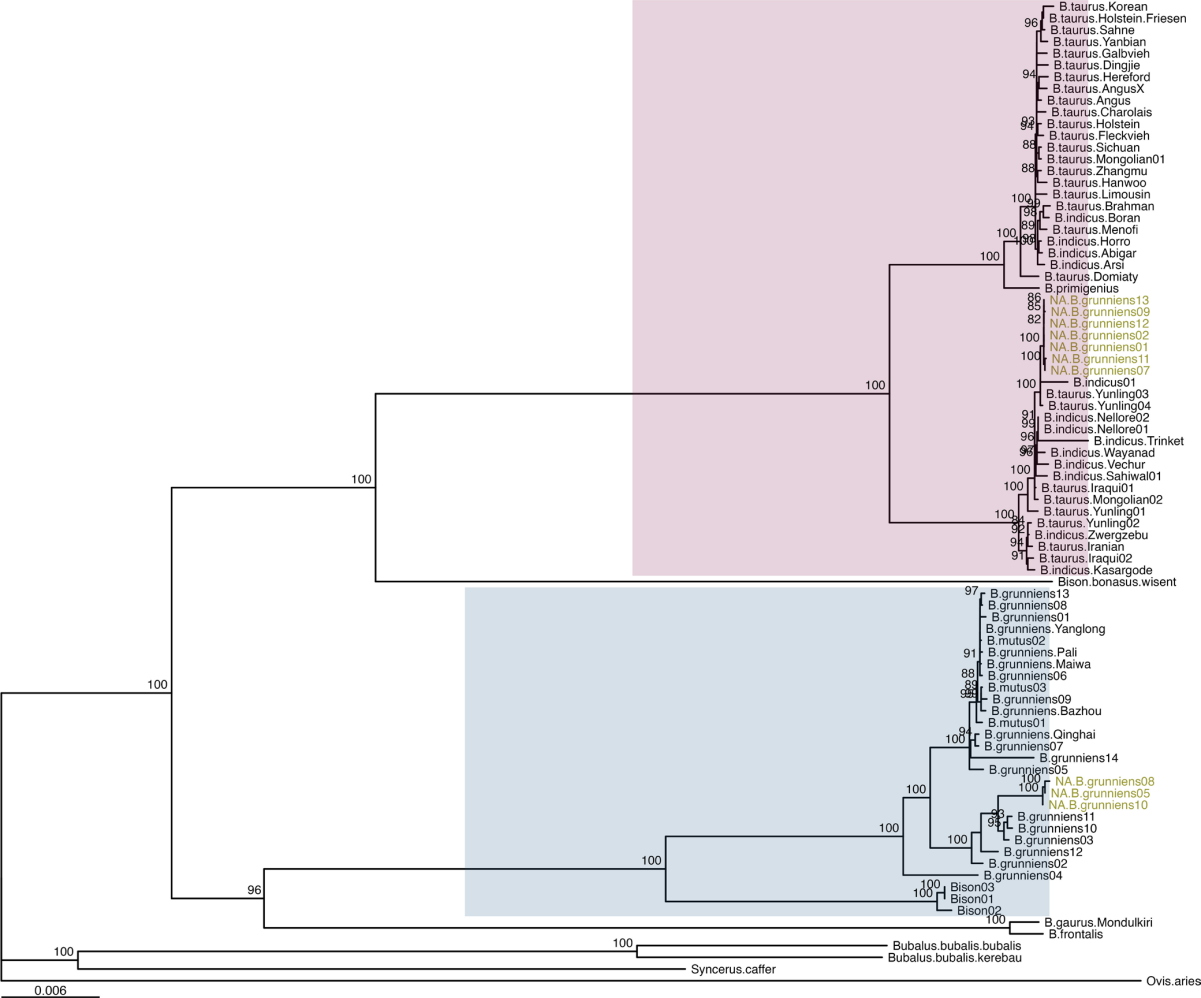
Phylogenetic tree based on complete mitochondrial sequences of Bovidae, including the North American yak (names shown in gold). Bootstrap values > 0.8 (80) are shown in the figure. *Ovis aries* was used as the outgroup. The cattle clade ( *B. taurus* and *B. indicus* ) is represented in pink and the yak clade is represented in blue. These clades have high confidence bootstrap values. NA *B. grunniens* 3 and NA *B. grunniens* 4 are not shown in the tree but share a mitotype with NA *B. grunniens* 1 and NA *B. grunniens* 2.

A maximum likelihood tree including the 12 NA yak, Asian domestic yak, and assorted Bovidae species separated the NA yak into two major clades supported by a bootstrap value of 1 (Figure 2). Seven NA yak mitotypes fell within one clade, while three NA mitotypes formed another clade. With high confidence, the first NA yak clade was placed near domestic cattle, while the other was most proximal to domestic yak.

### 3.3 Alignment to Asian Yak Breeds

After excluding the D‐loop, two of the NA yak mitotypes (IDs: NA *B. grunniens* 5 and NA *B. grunniens* 10) were identical to Haplotypes 63 and 107 of Wang et al. (2024), which originated from Jinchuan and Maiwa yak in the Sichuan Province of China. Haplotypes 63 and 107 of Wang et al. (2024) differed only by a single SNP in the D‐loop. The third NA yak (NA *B. grunniens* 8), while different from all others in the alignment, varied from the other two NA yak by only 4 bp.

### 3.4 Domestic Yak D‐Loop Network

**FIGURE 3:**
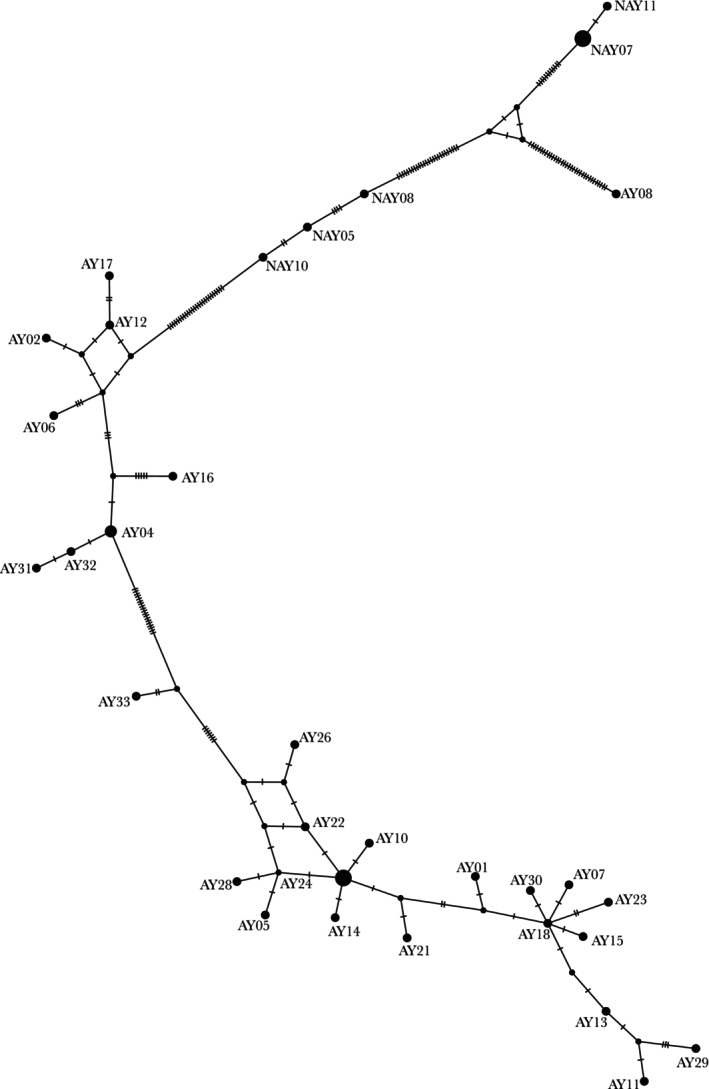
Median joining network of partial D‐loop sequences (835 bp) of Asian yak (AY) and North American yak (NAY). Mutations are represented by hatch marks. Three NA yak mitotypes fall next to the Asian yak, while the other two NA mitotypes, representing nine individuals, do not. AY08 represents an Asian yak with *B. taurus* mitochondria. While no *B. indicus* accessions were included in this haplotype network, there is a distinct separation between the *B. taurus* sequence and both NA yak groups.

Utilizing only D‐loop segments to compare the NA yak to 29 Asian domestic yak, the median joining network had 125 segregating sites, 92 of which were parsimony‐informative. The nucleotide diversity (*π*) was 0.041. We found five unique NA mitotypes, which were all divergent relative to the Asian yak lineage. No single mitotype was shared between any NA and Asian yak as visualized in the median joining network (Figure 3). One Asian yak, determined by Lai et al. (2007) to have a *B. taurus* mitotype, fell between the NA yak. Three mitotypes were close to the previously identified *B. grunniens* clade and two mitotypes of NA yak fell more proximal to domestic cattle.

### 3.5 Sanger Sequences

No additional variation was identified in the 3521 bp sequenced in the 17 additional NA yak. Nine of the 17 NA yak were classified as the yak‐type mitotype and eight as cattle‐type with no variation within either major mitotype.

### 3.6 Variant Functional Annotation

Variant Effect Predictor interpreted 111 of the 1116 variable sites as nonsynonymous (Table 1). Of the variants tested through the VEP, 499 represented existing variations as annotated by a RefSeq (rs) identifier.

**TABLE 1:** Summary of the number of variants identified in North American yak relative to the cattle reference genome (ARS‐UCD1.3; CM008198.1).

Gene	Total variants	Nonsynonymous
ND1	73	9
ND2	73	12
COX1	92	5
COX2	49	2
ATP8	15	5
ATP6	41	6
COX3	53	6
ND3	24	1
ND4L	23	2
ND4	114	16
ND5	157	30
ND6	40	4
CYTB	96	13
Dloop	96	N/A
tRNA/rRNA	170	N/A
Total	1116	111

### 3.7 Cell Isolation and Culture

No cattle introgression was detected in the autosomal genome of any yak from which satellite cells were derived as determined by the commercial introgression panel based on Kalbfleisch et al. (2020) (Neogen, Lincoln NE). Western blots for MyHC antibodies of the seven NA yak confirmed uniformity of fiber type across the samples. Cultures were over 87% pure on average as determined by PAX7 staining.

**FIGURE 4:**
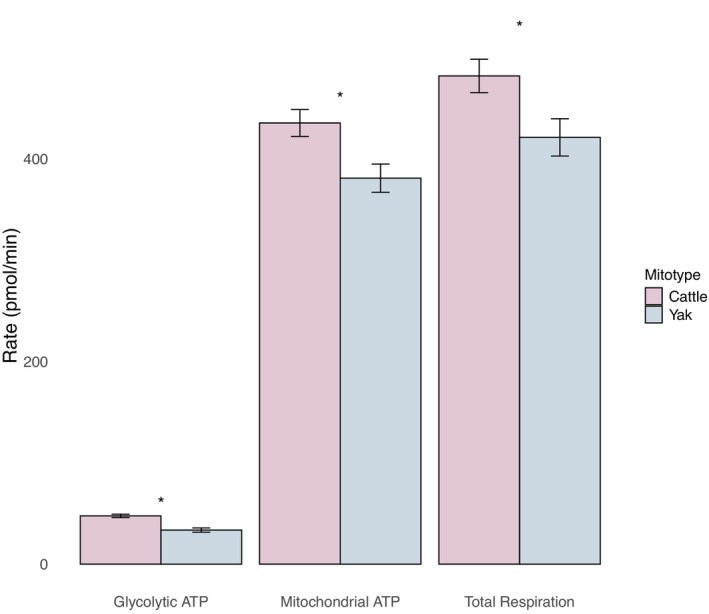
Mean respiration rates (pmol/min) as a measure of mitochondrial function. The two North American yak mitotypes (cattle *n* = 4, yak *n* = 3) differed in glycolysis (*p* = 0.014), oxidative phosphorylation (*p* = 0.018), and total respiration (*p* = 0.016). Mitochondrial ATP production made up the majority of the total respiration for both mitotypes. *indicates a significant difference (*p* = 0.05) between rates of ATP production between mitotypes.

After normalization for protein concentration, oxygen consumption rates analyzed by ANOVA for the effect of mitotype revealed that samples with cattle‐type mitochondria had a significantly greater rate of glycolysis (*p* = 0.014) and oxidative phosphorylation (*p*=0.018) than samples with yak mitochondria (Figure 4). There was no significant difference in the proportion of the ATP generated from each pathway by mitotype (*p* = 0.109) according to ANOVA analysis of the percent of total respiration generated by glycolysis or from oxidative phosphorylation. Total ATP production was 14% greater in samples with cattle‐type mitochondria.

## 4 Discussion

Complete mitochondrial sequences place NA yak into two distinct clades supporting two mitochondrial origins of the NA population. A haplotype network derived from partial D‐loop sequences also confirmed this division. These distinct clades had substantial genetic variation in protein‐coding genes, which we hypothesize contribute, in part, to the observed significant difference in total ATP production observed in satellite cells. This work serves to characterize the maternal origins and genetic identity of current NA yak herds and provides foundational information for further studies of how mitochondrial genotype affects its function.

In prior work, wild and domestic yak from Asia were both found within two major mitochondrial lineages, and more recently within a minor third lineage (Wang et al. 2021, 2024). Phylogenetic placement of NA yak classified them as having one of two mitotypes. One mitotype suggests shared ancestry and likely introgression from *B. indicus* cattle, while the other is closely associated with Asian yak in phylogenetic branches 2 and 3, haplogroups B and C as classified by Wang et al. (2021). The more recent publication of Wang et al. (2024) more specifically positions the NA yak of the latter mitotype with Jinchuan and Maiwa yak that were sampled in the Sichuan Province of China. This does not eliminate the possibility that NA yak not included in the study could represent other clades. At the time they were sequenced, however, the 12 NA yak were selected to represent as much diversity as possible based upon known ancestry and pedigrees. However, given the limited importation of yak to NA, and the small census size of the population, it is possible little additional variation exists.

Hybridization of yak with domestic cattle in NA has been, to our knowledge, limited to *B. taurus* breeds. Wiener et al. (2006) reported crosses of NA yak with cattle such as Galloway. In Canada and the United States, NA yak are rarely raised in the hot subtropical climatic regions where NA indicine cattle are common. Besides the outcome that NA yak did not have mitotypes similar to any taurine samples included in the analysis that are expected in NA domestic cattle, none of the NA yak had measurable autosomal cattle introgression. This implies either these yak were not previously crossbred, or that autosomal ancestry had been lost over time, leaving only mitochondrial ancestry indicative of prior introgression. Those lines of evidence, combined with the placement of the NA yak with samples representative of Asian breeds with indicine ancestry (e.g., Wayand, Vechur, Yunling [composite of Brahman, Murray Gray, Yunnan Yellow]), support that the introgression event observed occurred prior to importation to NA. The cattle with which the NA yak cluster closely represent cattle subgroup I1a, which is endemic to Chinese breeds (Xia et al. 2019). Given that result and the similarity of the NA yak of the other clade to yak from the Sichuan Province, China, these results may point to a potential origin of NA yak in that region. Crossing of cattle and yak occurs across Asia, although the impact on yak populations is low, in part due to the time scale at which these introgressions have occurred and the limited use of hybrids in breeding. For instance, Qi et al. (2010) demonstrated minimal or no detectable cattle introgression as quantified by autosomal loci in domestic yak with Asian *B. taurus* mitochondria. Additionally, autosomal variation associated with white coat color, coat color patterning, and nose pigmentation has also been attributed to crossing with cattle (Petersen et al. 2020; Zhang et al. 2023).

Comparing the *B. indicus* cattle and yak mitotypes identified in the NA yak, many nonsynonymous variants were observed in genes involved in oxidative phosphorylation, including some that were previously explored as potentially involved in high altitude adaptation (Shi et al. 2018; Wang et al. 2018). Physiological differences among yak and other Bovidae include expanded thoracic capacity (Leslie and Schaller 2009), nuclear‐encoded mitochondria genes (Wang et al. 2017), higher hemoglobin count, and lactate dehydrogenase levels (Kuang et al. 2010). It is possible that the efficiency or preferred mechanisms of energy production also differ between these species as an adaptation to their environment.

Variation between the two major mtDNA haplotypes in NA yak, involving genes critical to cellular metabolism, led to the hypothesis that the two mitochondrial haplotypes may functionally differ. This hypothesis was supported by the Seahorse assays demonstrating that the satellite cells with the cattle‐derived mitotype produced significantly more ATP via both glycolytic and oxidative metabolism. Although the sample size was small, this result suggests that variation in mtDNA could underlie functional differences in cellular metabolism leading to several hypotheses. For instance, modern cattle are generally provided a higher‐quality diet than what is available to yak in the QTP. This could favor energy production through glycolysis. However, since glycolysis is a less efficient pathway, having a larger proportion of ATP generated via oxidative phosphorylation could have a selective advantage in the yaks' traditional environment.

The results of this work provide insight into the diversity and origin of the mitochondrial genome in the NA yak. The low diversity observed supports the origin of the NA population from a small group of founders, while also providing evidence of past introgression with cattle of Asian origin. Improving the understanding of the diversity of the mitochondrial genome in the NA population benefits breeders as they plan the best management and selection programs for their herds and for the population overall. This study also reveals the first indication of functional differences between NA mitotypes and may contribute to future studies on mitochondrial evolution in high‐altitude adapted mammals.

## Author Contributions

**Leah K. Treffer:** formal analysis (lead), investigation (lead), visualization (lead), writing – original draft (lead). **Renae L. Schroeder:** formal analysis (supporting), investigation (supporting), methodology (supporting), supervision (supporting), writing – review and editing (supporting). **Edward S. Ricemeyer:** data curation (supporting), formal analysis (supporting), investigation (supporting), methodology (supporting), writing – review and editing (supporting). **Ted Kalbfleisch:** data curation (lead), methodology (supporting), resources (supporting), writing – review and editing (supporting). **Anna M. Fuller:** methodology (supporting), project administration (supporting), supervision (supporting), writing – review and editing (supporting). **Jessica L. Petersen:** conceptualization (lead), investigation (supporting), methodology (lead), project administration (lead), resources (lead), supervision (lead), writing – original draft (supporting).

## Ethics Statement

All samples utilized for the study were obtained directly from owners. As no live animal studies were conducted, a formal protocol was not necessary as per the requirements of the University of Nebraska‐Lincoln Institutional Animal Care and Use Committee.

## Conflicts of Interest

The authors declare no conflicts of interest.

## Supporting information


**Table S1:** ece372362‐sup‐0001‐TableS1.xlsx.

**Table S2:** ece372362‐sup‐0002‐TableS2.xlsx.

**Table S3:** ece372362‐sup‐0003‐TableS3.xlsx.

**Data S1:** ece372362‐sup‐0004‐DataS1.docx.

## Data Availability

The whole‐genome sequence generated for the 12 North American yak is available at: https://www.ars.usda.gov/plains‐area/clay‐center‐ne/marc/wgs/artiobov/yak/ with IDs of the yak used in this study matched to the public repository available in Table S3. To access these data, the bam files can either be downloaded directly via wget or curl on high performance computers. To obtain the reads mapped to the CM008198.1 mitochondrial sequence via samtools: samtools view *pastedURL* ‐hb > output.bam. Sanger sequence data generated is available in GenBank (Accession PX434797‐PX434806).
