# Supplementary material for: Mitochondrial Introgression With Potential Functional Effects in North American Yak
Source: Ecol Evol. 2025 Oct 18;15(10):e72362. doi: 10.1002/ece3.72362 (PMC12535201; doi:10.1002/ece3.72362)
Supplement: Supplementary file 4 — Data S1: ece372362‐sup‐0004‐DataS1.docx. [file ECE3-15-e72362-s001.docx]

**Supplemental Protocols: Mitochondrial Introgression with Potential Functional Effects in North American Yak**

Leah K. Treffer, Renae L. Schroeder, Edward S. Ricemeyer, Ted Kalbfleisch, Anna M. Fuller, Jessica L. Petersen

**Sanger sequencing protocol**

PCR primers were designed using Primer3 (Untergasser et al. 2012). PCR reactions consisted of (per reaction) 1.2µl 10x PCR Buffer, 0.5µl dNTP (10mM each), 0.1µl of Taq polymerase (5 U/µl; FastStart, Sigma-Aldrich), 0.75µl of 20µM forward and reverse primer (Table SP1), template DNA, and water to 12µl final volume.

| Loci | Primer Name | Sequence (5' to 3') | Fragment Coordinates (CM008198.1) | Fragment Size (bp) | Annealing Temp (°C) | DNA Template/Rxn |
| --- | --- | --- | --- | --- | --- | --- |
| tRNA-Gln, tRNA-Met, ND2, tRNA-Trp, tRNA-Ala | Bovine_ND2_F1 | CCAAAACTCTTCGTGCTCCC | 4158-4822 | 665 | 58 | 15ng |
|  | Bovine_ND2_R1 | TGAGCGATTGATGAGTAGGCT |  |  |  |  |
|  | Bovine_ND2_F2 | GGCCTTATCCTACTGACATGAC | 4644-5398 | 755 | 58 | 15ng |
|  | Bovine_ND2_R2 | AAGGTGTAGTCTTGCAATCCTT |  |  |  |  |
| CTYB, tRNA-Glu, ND6, ND5 | Bovine_ND6_F1 | AGTGGGTGGGACTTTCGAAT | 13824-14542 | 719 | 54 | 10ng |
|  | Bovine_ND6_R1 | AGCATCTACCCTGGTCACAA |  |  |  |  |
| ATP8, ATP6 | Bovine_ATP6_F1 | CCGCAACTAGACACGTCAAC | 8132-8831 | 700 | 58 | 15ng |
|  | Bovine_ATP6_R1 | AGTGCAAGTGTAGCTCCTCC |  |  |  |  |
| ND4L, ND4 | Bovine_ND4_F1 | AGCAGCCCTAACAATCCTCA | 10367-11048 | 682 | 58 | 20ng |
|  | Bovine_ND4_R1 | GGGATCCTACTGTGTTTTGGA |  |  |  |  |

Table SP1. Sanger sequencing primers, loci included and thermalcycling information.

Thermalcycling included an initial denaturation of 4 min at 94°C, followed by 32 cycles of 94°C for 30sec, annealing temp (Table SP1) for 30sec, and a 72°C extension for 45 s. Following a final 10min extension at 72°C, products were held at 10°C.

3µl of PCR product was prepared for sequencing by adding 0.75µl Exosap and 13.25µl molecular water. The samples were incubated first at 37°C for 30 min followed by 15 min at 80°C prior to a 15°C hold. Sequencing was performed by ACGT INC (Wheeling, IL) using both the forward and reverse primers.

**Western blot protocol**

A 100mg muscle sample, stored in RNAlater (Invitrogen) at the time of harvest were homogenized and sonicated at 30% amplitude in 300 ul Pierce RIPA buffer containing Halt Protease and Halt Phosphatase Inhibitors (Thermo Fisher Scientific). Suspensions were centrifuged at 14,000 x g for 5 min at 4°C. Total protein was quantified with a Pierce BCA Protein Assay (Thermo Fisher Scientific).

Protein was incubated at room temp for 10 min at 1X with a 4X Laemmli Sample Buffer (Bio-rad) and β-mercaptoethanol, then denatured at 70°C for 10 min. In each well, 30 µg protein was separated by SDS-PAGE (Yates et al., 2016). After electrophoresis on a Mini-PROTEAN Tetra Cell at 4°C for 24 h at a constant 110 V, gels were semi-dry transferred to a LF PVDF membrane at 2.5 A for 15 min (Bio-rad). Revert 700 Total Protein Stain (Li-Cor) was used to stain the membrane for total protein. Membranes were blocked in EveryBlot Blocking Buffer (Bio-rad) at room temperature for 10 min. After blocking, membranes were incubated with a MyHC types I and IIA antibody (DSHB, AB_10573811) at a 1:100 dilution (0.32 ug/mL final concentration) or MyHC type I and IIX antibody (DSHB, AB_2314830) at a 1:500 dilution (38 ng/mL final concentration). IRDye 800CW goat anti-mouse IgM secondary antibody (Li-Cor, AB_2814919) at a 1:10,000 dilution and a Li-Cor Odyssey infrared imaging system were used to visualize immunoreactivity. Blots were analyzed using Image Studio Lite Software version 5.2 (Li-Cor).

**References**

Untergasser A, Cutcutache I, Koressaar T, Ye J, Faircloth BC, Remm M and Rozen SG. Primer3--new capabilities and interfaces. Nucleic Acids Res. 2012 Aug 1;40(15):e115.

Yates DT, Cadaret CN, Beede KA, Riley HE, Macko AR, Anderson MJ, Camacho LE, Limesand SW. 2016. Intrauterine growth-restricted sheep fetuses exhibit smaller hindlimb muscle fibers and lower proportions of insulin-sensitive Type I fibers near term. American Journal of Physiology-Regulatory, Integrative and Comparative Physiology. 310(11):R1020-1029. https://doi.org/10.1152/ajpregu.00528.2015.
